# Clonotype definitions for immunogenetic studies: proposals from the EuroClonality NGS Working Group

**DOI:** 10.1038/s41375-023-01952-7

**Published:** 2023-06-30

**Authors:** Electra Sofou, Elisavet Vlachonikola, Laura Zaragoza-Infante, Monika Brüggemann, Nikos Darzentas, Patricia J. T. A. Groenen, Michael Hummel, Elizabeth A. Macintyre, Fotis Psomopoulos, Frederic Davi, Anton W. Langerak, Kostas Stamatopoulos

**Affiliations:** 1 Institute of Applied Biosciences, Centre for Research and Technology Hellas, Thessaloniki, Greece; 2 Department of Hematology, University Hospital Schleswig-Holstein, Kiel, Germany; 3 Department of Pathology, Radboud University Medical Center, Nijmegen, The Netherlands; 4 Institute of Pathology, Charité-Universitätsmedizin Berlin, Berlin, Germany; 5 Department of Hematology, Université Paris Cité and APHP Necker-Enfants Malades, Paris, France; 6 Hematology Department, Hospital Pitié-Salpêtrière and Sorbonne University, Paris, France; 7 Department of Immunology, Laboratory Medical Immunology, Erasmus MC, University Medical Center Rotterdam, Rotterdam, The Netherlands

**Keywords:** Preclinical research, Lymphoid tissues

## To the Editor:

Comprehensive study of immunoglobulin (IG) and T cell receptor (TR) gene rearrangements has proven instrumental for understanding immune responses in health and disease, while also offering information with direct clinical utility e.g., for minimal residual disease detection or clonality assessment in patients with lymphoid malignancies [1]. However, immunogenetic analysis entails descriptive definitions that are often arbitrary.

A particular challenge is posed by the term “clonotype”, generally referring to a unique antigen receptor gene rearrangement, for which different definitions have been proposed (Supplementary Table 1). The lack of a consistent definition of what a clonotype is can lead to different interpretations. This is especially true in Next Generation Sequencing (NGS) repertoire studies, where clustering of rearrangement sequences to clonotypes happens at the initial stages of data processing, thus affecting (meta)data interpretation. That said, it is crucial to reach a consensus on the stringent definition of a clonotype while highlighting cases where an alternative definition could be appropriate, depending on a given context or specific research hypothesis.

**Fig. 1:**
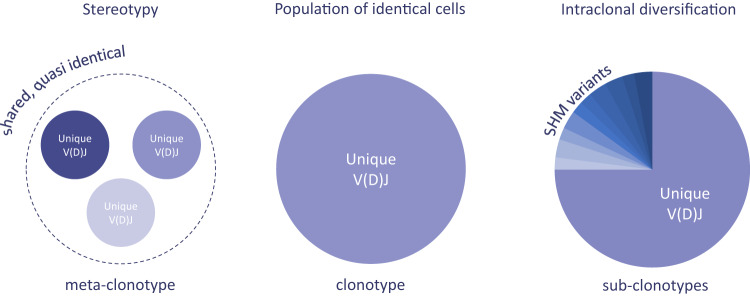
Clonotype definitions. The stringent definition of the term clonotype is a unique antigen receptor gene rearrangement nucleotide sequence corresponding to a population of identical cells. Sub-clonotypes arise in the context of intraclonal diversification within IG genes due to the presence of SHM variants. Meta-clonotypes can be defined in the context of stereotypy, referring to unrelated clones sharing (quasi)identical VDJ rearrangements.

Since each antigen receptor corresponds to a given B/T cell, strictly speaking, the term “clonotype” should correspond to a unique V(D)J nucleotide sequence (Fig. 1). This definition becomes more complicated when moving from single cells to clones of cells. In the latter case, derivation from a single ancestral cell means that, in principle, all clonal cells should share the same clonotype. However, in reality, variations exist leading to (sub)clonal heterogeneity: by extension this raises the need for “context-specific” definitions. Relevant examples are outlined below.

Different B/T cell clones in the same or unrelated individuals may carry quasi-identical, “stereotyped” BcR IG or TR.

BcR IG stereotypy has been documented in normal B cells but also in the context of vaccination and infections [2] or mature B-cell malignancies [3, 4]. In the context of stereotyped BcR IG, clonotypes should be considered as clusters of sequences from independent B-cell clones that are grouped together based on V gene and CDR3 identity/similarity, which herein we propose to be defined as “meta-clonotypes” (Fig. 1). Although this approach considers different clonοtypes as one, thereby reducing the complexity of a given repertoire, it can be biologically and clinically relevant, e.g., for refining patient risk stratification [3, 4] (also see Supplementary Material).

Stereotyped TRs have been reported in different contexts, unsurprisingly on a shared HLA background [5], extending from infections to graft-versus-host-disease, anti-tumor responses or T-cell lymphoproliferations (e.g., T-LGL leukemia, etc) [6]. In some relevant examples, the common TR alpha or beta chain CDR3 shared by different T-cell clones/patients may be encoded degenerately by different nucleotide sequences (“convergent recombination”) [7], however, it would be amiss to not cluster these sequences in a single, “archetypical” clonotype (Supplementary Fig. 2).

**Fig. 2:**
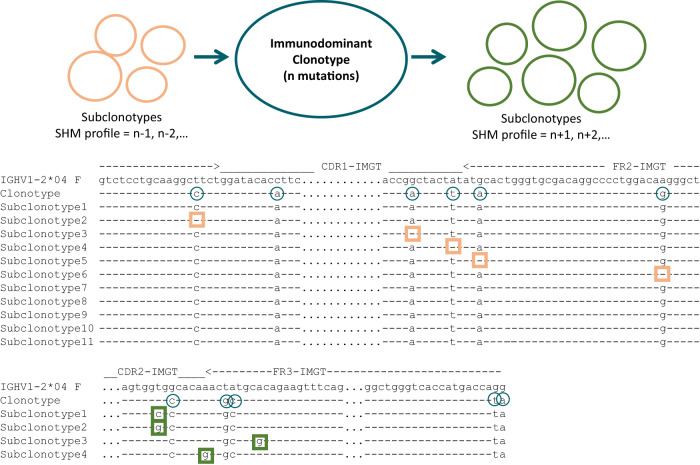
Intraclonal diversification due to ongoing SHM. Example of a SMZL case expressing an IGHV1-2*04/IGHD3-3*01/IGHJ4*02 gene rearrangement. Alignment of nucleotide sequences that express the same AA VH CDR3 however present with different SHM profiles in the IGHV nucleotide sequence (“subclonotypes”).

Whilst stereotypy stands at one end of the spectrum of repertoire diversity, in B cells, the other end of the spectrum is represented by intraclonal diversification (ID) within the rearranged IG genes due to ongoing somatic hypermutation (SHM) [8] or due to aberrant (antigen-independent) introduction of SHM as present in some lymphomas [9]. When studying ID, the aim is to accurately map the complexity of the sub-clonal architecture: in such cases, only stringent criteria for defining a clonotype are appropriate. For instance, even when the V gene and the CDR3 nucleotide sequences are identical, yet the SHMs in the FR1-FR3 part of the V domain differ, the respective IGHV-IGHD-IGHJ rearrangement nucleotide sequences should be considered as distinct clonotypes’, thus proposing the term “sub-clonotypes”; (Figs. 1, 2) despite that they originate from the same unique founder cell.

ID may also occur in malignancies of immature lymphoid cells. A prime example is B-acute lymphoblastic leukemia (B-ALL) where multiple new but related IG rearrangements may result from RAG-mediated recombination that may still be active in the malignant clone [10, 11]. In this context, the term clonotype can also be utilized for partial and/or unproductive rearrangements which may not encode for a functional antigen receptor chain yet constitute another unique molecular identity for a given B/T-cell clone.

The ever-increasing application of NGS in hemato(patho)logy poses a significant challenge regarding the interpretation of the findings concerning the size of the clonal cell population. For example, when assessing the clonality or MRD status based on BcR IG gene repertoire analysis by NGS, one has to take into account sequence divergence throughout the variable domain, including the VH CDR3 [12]. In this case, loosening the criteria for defining a clonotype helps to fully capture all the ‘branches’ of the same clonal population. To that purpose, one could compute clonotypes not as unique V(D)J nucleotide sequences but instead as clusters of sequences with the same V gene and VH CDR3s of identical length displaying high (minimum 80%) identity in the amino acid sequence.

Another important issue concerns unproductive or partial IG gene rearrangements and rearrangements involving the kappa deleting element (KDE) that lead to the inactivation of the respective IGK locus [13, 14]. These may not encode for a productive antigen receptor chain yet represent clonal signatures and should be reported as such.

Besides the biological heterogeneity, there are also technical aspects that need to be considered when defining clonotypes in a diagnostic setting, since they may falsely impact clonal size estimation. In detail:

- TR gene rearrangements do not carry SHM. By logical extension, variations in the TR gene rearrangement sequence have to be attributed to PCR and/or sequencing errors: they can be ignored biologically but should still be included in the computation of the T-cell clone size (Supplementary Fig. 2).
- Intraclonal variations can be observed in sequences sharing identical V, D and J genes and identical amino acid junctions, although with differences in the alignment of the NDN regions due to PCR or sequencing errors. In such cases, all relevant sequences should be considered as belonging to a single clonotype.

It is worth mentioning that when working with suboptimal starting material, such as DNA extracted from formalin-fixed, paraffin-embedded tissues or liquid biopsies, smaller fragments may be obtained. More generally, small PCR amplicons may also arise if PCR amplification is performed using FR2 or FR3 primers. This issue may lead to misalignment of the IGHV/TRBV genes on the germline sequences, eventually causing generic assignments to different genes. Hence, clonotype computation and clonality assessment should be carefully handled, as both over- and under-estimation of the clonal size could occur.

The EuroClonality-NGS Working Group presents here the following definitions for the terms *clonotype*, *meta-clonotype* and *sub-clonotype*:

- *Clonotype*: a unique V(D)J nucleotide sequence
- *Meta-clonotype*: clusters of sequences from independent B/T-cell clones that are grouped together based on V gene and CDR3 identity/similarity (antigen receptor stereotypy and convergent recombination)
- *Sub-clonotype*: rearrangement sequences using the same V gene and identical CDR3 nucleotide sequences, yet with SHMs that may differ in the FR1—FR3 part of the V domain.

Our aim is to promote accurate interpretation of immunogenetic cues in both diagnostics and research through the adoption of a common glossary that will enable fully harnessing the available information.

## Supplementary information

Supplemental Material
