# Supplementary material for: Clonotype definitions for immunogenetic studies: proposals from the EuroClonality NGS Working Group
Source: Leukemia. 2023 Jun 30;37(8):1750–2. doi: 10.1038/s41375-023-01952-7 (PMC10400411; doi:10.1038/s41375-023-01952-7)
Supplement: Supplementary file 1 — Supplemental Material [file 41375_2023_1952_MOESM1_ESM.docx]

**Supplementary material**

**Supplementary table 1.** Different clonotype definitions utilized by independent platforms dedicated to immunogenetics analysis(1–4).

| **Platform** | **Clonotype definition** | **Reference** |
| --- | --- | --- |
| Vidjil | k-mers ranging from 9 to 13nt corresponding from V and J regions on each read, allowing to locate a “window” overlapping the actual CDR3 | Duez et al. PLoS One. 2017 |
| ARResT/Interrogate | V, D and J genes and alleles that are combined with the amino acid sequence of the CDR3 | Bystry et al. Bioinformatics 2017 |
| immunoSEQ Analyzer (Adaptive Biotechnologies) | The count of unique rearrangements in nucleotide level within a sample that are generated through V(D)J recombination | https://www.immunoseq.com/analyzer/ |
| IMGT/StatClonotype | Unique V-(D)-J rearrangement (with IMGT gene and allele names determined by IMGT/HighV-QUEST at the nucleotide level) and a unique CDR3-IMGT amino acid (in-frame) junction sequence | Aouinti et al. Front. Immunol. 2016 |
| MiXCR | CDR3 nucleotide sequence | Bolotin et al. Nat Methods 2015 |

**Clonotypes in the context of stereotyped B cell receptor immunoglobulin: the case of CLL Stereotyped subset #2**

Patients with chronic lymphocytic leukemia assigned to stereotyped subset #2 express B cell receptor immunoglobulin (BcR IG) encoded by the IGHV3-21/IGLV3-21 gene pair with highly (quasi)identical VH and VL CDR3s. In the case of subset #2, cases with identical VH CDR3 amino acid sequence could be considered as representing a single, “archetypical” clonotype (Suppl. Figure 1A), despite differing in the rest of the variable domain due to distinct SHM (Suppl. Figure 1B). However, despite such differences, patients in subset #2 share clinical manifestations and outcomes, including the response to therapy(5), thus justifying the application of more relaxed criteria for clonotype definition and computation.(6)


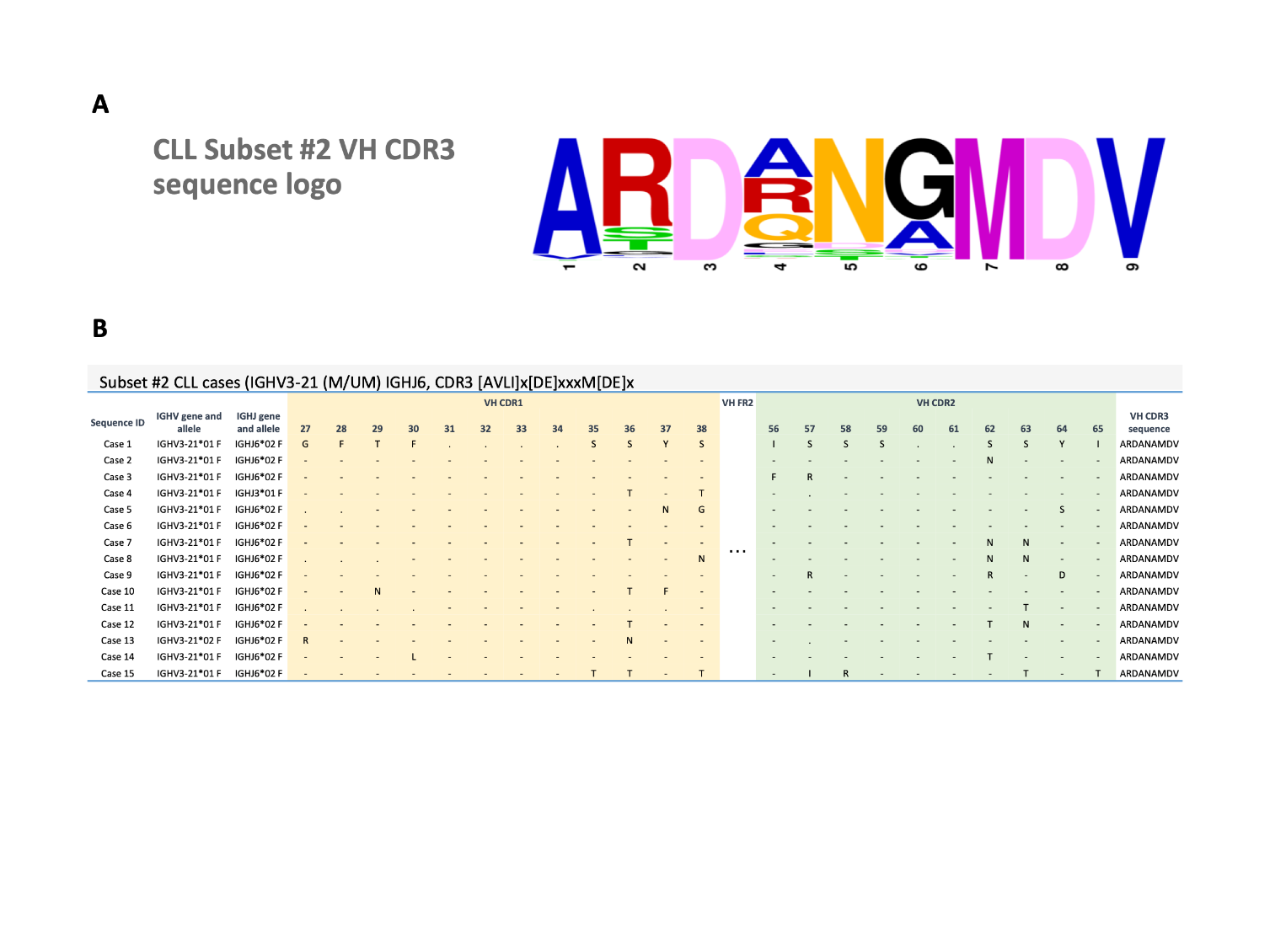


**Supplementary Figure 1. Defining clonotypes in the context of BcR IG stereotypy.** VH CDR3 AA sequence logo of CLL stereotyped subset #2 cases utilizing the IGHV3-21/IGLV3-21 gene pair (A). Different cases expressing the same IGHV gene and identical VH CDR3 amino acid (AA) sequence (forming a “meta-clonotype”) display different SHM patterns over the VH CDR1 and CDR2 (B).

**Supplementary Figure 2**
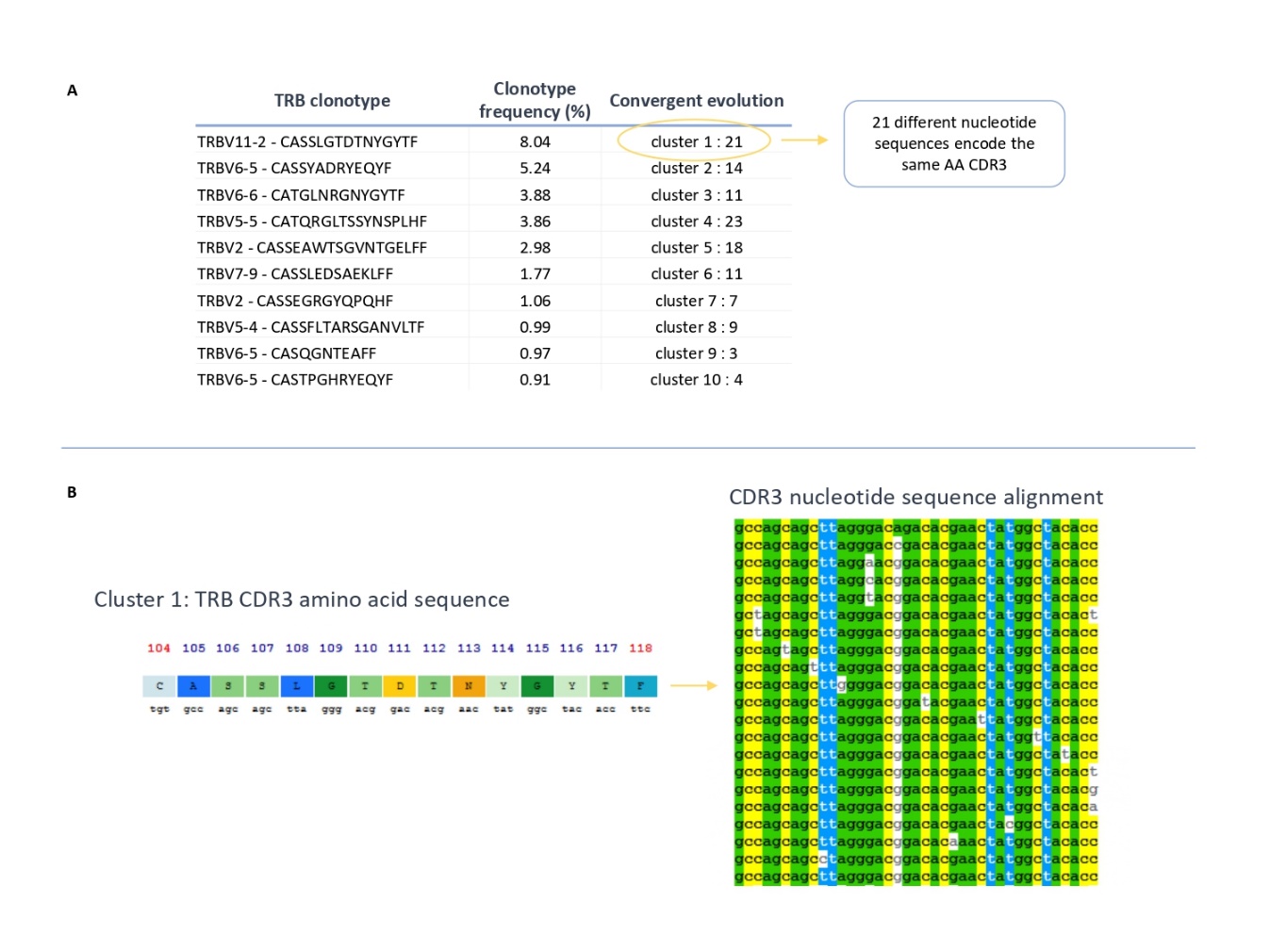

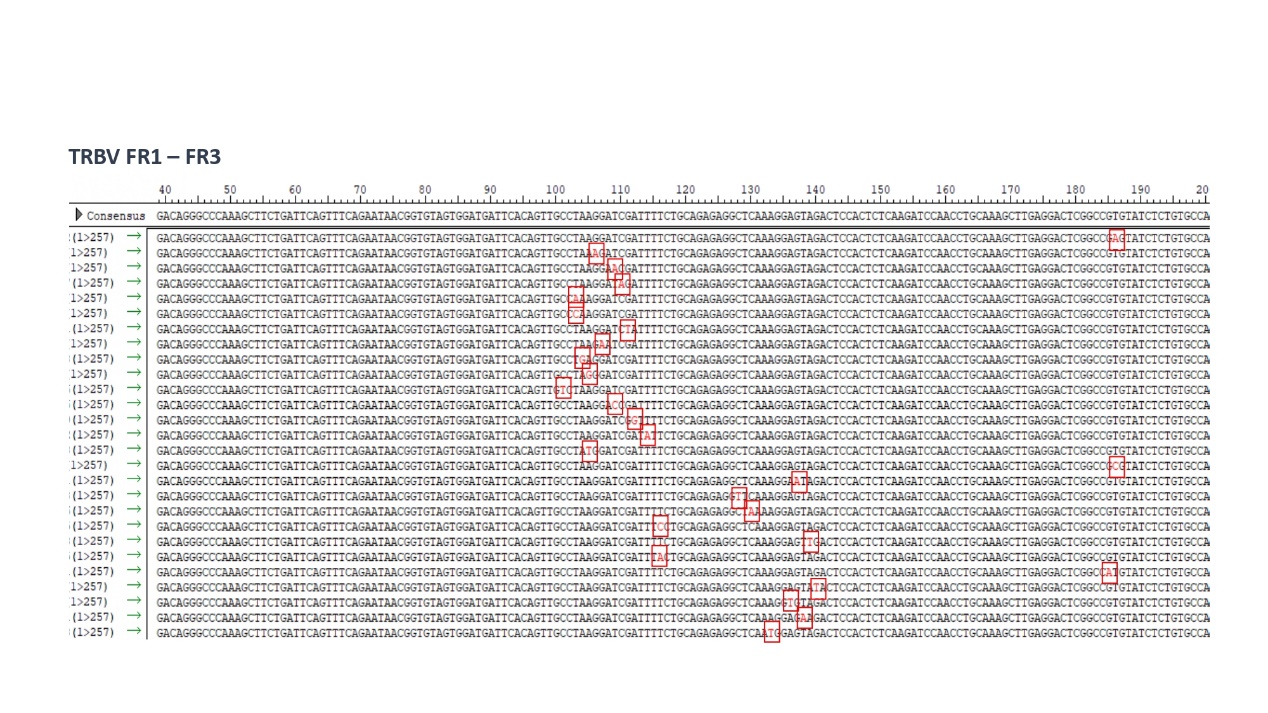


**C**

**Supplementary Figure 2**: **Convergent recombination.** Illustrative case of clonality assessment in a 67-year-old male with clinical and immunophenotypic evidence of T-LGL lymphoproliferation. The major TRBV AA sequence was encoded by 21 different TRBV CDR3 nucleotide sequences (A-B). Possible explanations in this case include convergent recombination (biological explanation) but also PCR/sequencing artefacts (technical explanation); the latter is strongly supported when nucleotide differences are observed over FR1-FR3 (C), which cannot be attributed to anything else but errors (SHM does not occur in TR rearrangement sequences).

**References**

1. Bystry V, Reigl T, Krejci A, Demko M, Hanakova B, Grioni A, et al. ARResT/Interrogate: an interactive immunoprofiler for IG/TR NGS data. Bioinformatics [Internet]. 2016 Oct 13 [cited 2020 Dec 27];33(3):btw634. Available from: https://pubmed.ncbi.nlm.nih.gov/28172348/

2. Aouinti S, Giudicelli V, Duroux P, Malouche D, Kossida S, Lefranc MP. IMGT/StatClonotype for Pairwise Evaluation and Visualization of NGS IG and TR IMGT Clonotype (AA) Diversity or Expression from IMGT/HighV-QUEST. Front Immunol [Internet]. 2016 Sep 9 [cited 2022 Jan 5];7(SEP). Available from: http://journal.frontiersin.org/Article/10.3389/fimmu.2016.00339/abstract

3. Bolotin DA, Poslavsky S, Mitrophanov I, Shugay M, Mamedov IZ, Putintseva E v, et al. MiXCR: software for comprehensive adaptive immunity profiling. Nat Methods [Internet]. 2015 May 29 [cited 2022 Jan 5];12(5):380–1. Available from: http://www.nature.com/articles/nmeth.3364

4. Duez M, Giraud M, Herbert R, Rocher T, Salson M, Thonier F. Vidjil: A Web Platform for Analysis of High-Throughput Repertoire Sequencing. PLoS One. 2016 Nov 11;11(11):e0166126.

5. Sonia Jaramillo, Andreas Agathangelidis, Christof Schneider, Jasmin Bahlo, Sandra Robrecht, Eugen Tausch, et al. Prognostic impact of prevalent chronic lymphocytic leukemia stereotyped subsets: analysis within prospective clinical trials of the German CLL Study Group (GCLLSG). Haematologica. 2019 Dec 26;105(11):2598–607.

6. Gemenetzi K, Psomopoulos F, Carriles AA, Gounari M, Minici C, Plevova K, et al. Higher-order immunoglobulin repertoire restrictions in CLL: the illustrative case of stereotyped subsets 2 and 169. Blood. 2021 Apr;137(14):1895–904.
